# Diagnosis of Hereditary Syphilis

**Published:** 1894-01

**Authors:** 


### Diagnosis of Hereditary Syphilis.

Fournier insists that the diagnosis of hereditary syphilis must be based almost exclusively upon ocular evidence. Beyond a family history of infantile polymortality, verbal information has but little significance. To Hutchinson's triad of symptoms—

notched teeth, interstitial keratitis, and inflammation of the middle ear—must be added three other important evidences of specific trouble that do not always receive sufficient emphasis. First, the general appearance of the patient; next, the cicatrices upon the skin and mucous membranes; and, third, the deformities of the bones. Persistent childhood best decribes the bodily condition, owing to the low stature and slight frame. The scars upon the skin and mucous membranes present four definite characteristics: their size, configuration, area and locality. They are very small, almost minute; in form like a rounded bow, an arcade, or a serpentine curve. They are arranged in groups joined at the side, and are found at the commissure of the lips, about the circumference of the nose, in the throat, and particularly upon the buttocks and in the lumbar region. In the bony skeleton are the most strongly marked, the most profound and persistent lesions, easily discovered in the skull, in the face, in the bones of the extremities, most notably in the tibia. The forehead bulges outward, or may be keel-shaped, or may present symmetrical protuberance. There is often marked increase in the transverse diameter of the head, and the lateral halves of the cranium often show marked asymmetry. The characteristic deformity of the face is that of the nose. In the first type the upper part of the nose seems to have fallen in, and the tip turns up; in the second, the lower portion of the nose is shortened, and appears in some way to be absorbed into the upper part. There are also less typical nasal deformities, as when the nose is flattened at the base or badly formed, the result of simple dystrophy, and not of necrosis. Protuberances and hyperostoses are found at the level of the epiphyses, especially noticeable at the upper extremity of the tibia and the lower extremity of the radius and ulna. But the tibia is the direct revelation of syphilis. Its form alone will reveal this disease. It is large, thick, and misshapen, much widened and depressed in its middle portion. The crest is so much increased in size as to present a plane face. In general effect the bone is like the blade of a sword. This conformation is never found in any condition other than hereditary syphilis, of which it is the pathognomonic sign. Chronic lesions of the testicle are of frequent occurrence.

This, together with a history of middle-ear trouble, interstitial keratitis, notched teeth, small scars upon the skin and mucous membrane, the imperfections of the bony skeleton, the arrest or anomalies of bodily development, and the special condition of the tibia, render the retrospective diagnosis of hereditary syphilis comparatively easy. —*N. Y. Medical Record.*

